# Study on the Photothermal Performance Enhancement of Direct Absorption Solar Collectors by Heat-Transfer-Oil-Based MWCNT-TiN Composite Nanofluids

**DOI:** 10.1021/acsomega.6c04534

**Published:** 2026-10-15

**Authors:** Jing Hu, Dehong Gong, Jianguo Yan, Changyang Peng, Guomin Gan, Yuhang Xie, Zheng Jian

**Affiliations:** † Electrical Engineering College, 670212 Guizhou University, Guiyang 550025, China; ‡ Power China Guizhou Engineering Co., Ltd., Guiyang 550003, China

## Abstract

To improve the volumetric solar absorption capability of the working fluid in direct absorption solar collectors (DASC) and to extend its application to medium- and high-temperature solar-thermal utilization, mono multiwalled carbon nanotube (MWCNT) nanofluids and MWCNT-TiN hybrid nanofluids were prepared using Therminol 66 heat-transfer oil as the base fluid by a two-step method. Unlike previous MWCNT-TiN studies mainly based on aqueous systems, this work introduces the hybrid absorber into a low-polarity heat-transfer oil medium and employs oleic-acid-assisted dispersion and ultrasonication to improve particle dispersion in the oil phase. The optical and photothermal conversion performances of MWCNT nanofluids were first evaluated. The results show that the addition of MWCNTs substantially enhances solar absorption; the average transmittance of the nanofluid decreased from 68.43% for the base fluid to 33.44%, and the maximum apparent photothermal conversion efficiency reached 82.51% at 10 ppm MWCNT. TiN nanoparticles were then introduced into the optimized MWCNT nanofluid to prepare MWCNT-TiN hybrid nanofluids. The hybrid nanofluids exhibited further enhanced absorption in the visible–near-infrared range. At a TiN concentration of 10 ppm, the MWCNT-TiN hybrid nanofluid achieved the highest apparent efficiency of approximately 88.48%, which was 5.97 percentage points higher than that of the 10 ppm MWCNT nanofluid. This enhancement is mainly attributed to the strengthened broadband absorption in the visible–near-infrared region after TiN incorporation; this behavior is consistent with the reported plasmonic absorption characteristics of TiN, although the efficiency improvement is not attributed solely to a single LSPR mechanism. Supplementary stability evaluation of the optimized 10 ppm MWCNT–10 ppm TiN nanofluid showed that the UV–vis absorbance retention after 10 and 20 thermal cycles was approximately 98.0% and 95.5%, respectively. The particle-size distribution showed a Z-average of 66.533 nm, a PDI of 0.125, and a main peak at 75.529 nm, indicating good short-term optical and dispersion stability. This study provides a reference for the selection and application of oil-based hybrid nanofluids in medium- and high-temperature DASC systems.

## Graphical abstract

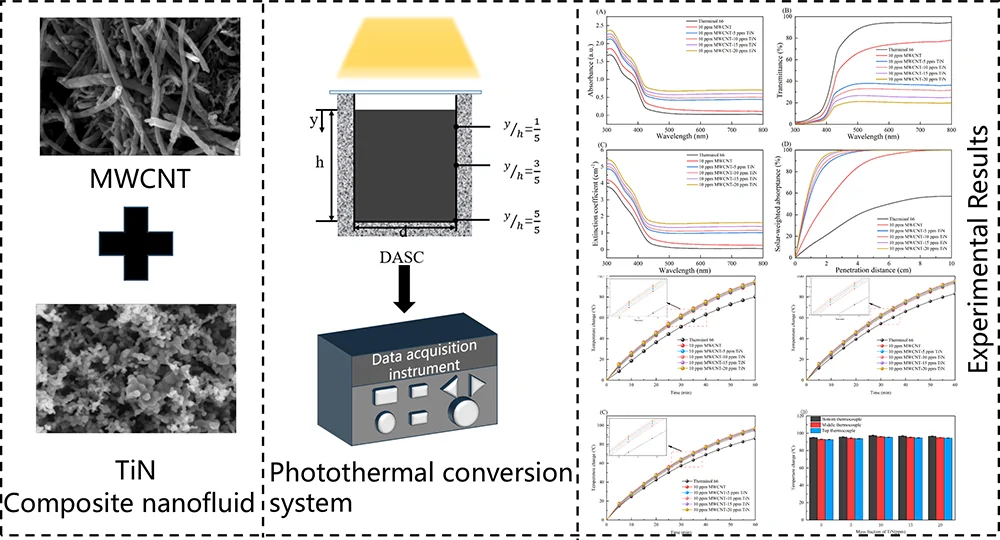

## 1 Introduction

Under the carbon-neutrality vision, solar energy has attracted increasing attention because it is clean, renewable, and capable of both photovoltaic and photothermal utilization, making it an important support for energy security and the optimization of the energy structure. Smalley proposed the “terawatt challenge” in 2005, calling for 30–60 TW of carbon-free energy by 2050 to sustainably meet global demand.^1^ In 2024, the installed solar capacity reached approximately 1.5 TW, representing an important step toward Smalley’s original target. Solar collectors are key devices for solar-thermal utilization because they capture solar radiation and convert it into thermal energy. Conventional collectors rely on black surface coatings to absorb radiation and then transfer heat to the heat-transfer fluid (HTF) flowing inside the tube.^2^ Because of the thermal resistance associated with indirect heat transfer, further improvement in system efficiency is limited. Direct absorption solar collectors (DASC) were therefore proposed^3^ to reduce energy losses in the heat-transfer process by allowing the working fluid to absorb solar energy directly and volumetrically. However, conventional working fluids, such as water, ethylene glycol, and heat-transfer oils, generally show weak solar absorption, which greatly limits DASC efficiency.^4,5^ In this context, the development of new working fluids has become a research focus. By dispersing nanoparticles into base fluids, nanofluids can effectively improve the optical absorption and heat-transfer capability of DASC working fluids.^6,7^

From a broader thermal-energy-system perspective, nanofluids and hybrid nanofluids are not limited to DASC but have also been widely explored as heat-transfer enhancement fluids in heat exchangers, thermoelectric refrigeration, electronic cooling, ground-source heat pumps, thermal energy storage, and solar-thermal systems. Their enhancement mechanisms mainly include increasing the effective thermal conductivity and thermal diffusivity of the base fluid through highly conductive solid nanoparticles, enhancing local heat transfer through Brownian motion, particle–fluid interfacial energy transport, and near-wall disturbances, improving the heat-transfer coefficient and Nusselt number in convective systems, and increasing the extinction coefficient and volumetric absorption capability in photothermal systems.^8,9^ Compared with mono nanofluids, hybrid nanofluids combine two or more types of nanoparticles to synergistically regulate thermal conductivity, optical absorption, stability, magnetic/electrical response, or cost, thereby achieving a more suitable overall performance for specific engineering scenarios.^9^ Recent system-level studies further indicate that nanofluid evaluation should extend from single thermophysical-property enhancement to practical system energy consumption, operational stability, and techno-economic and environmental benefits. For example, Cuce et al^10^ used Al_2_O_3_–TiO_2_–SiO_2_/water hybrid nanofluids as hot-side coolants in portable thermoelectric refrigerators and showed that hybrid nanofluids can improve thermoelectric refrigeration performance by enhancing Peltier hot-side heat dissipation. Another thermoelectric refrigeration study showed that, compared with pure water cooling, nanofluid cooling allowed the refrigerated cabinet to reach the target temperature faster, thereby helping reduce the operating time and electricity consumption required to achieve the same cooling effect. Furthermore, when CuO–Al_2_O_3_/water hybrid nanofluids were used in thermoelectric vaccine refrigerators, the time required to reach the target temperature of 4 °C was reduced from 1200 to 990 s, while the energy consumption and carbon emissions over a 15-year life cycle decreased by 18.3% and 12.3%, respectively.^11^ For high-heat-flux electronic cooling, hybrid nanofluid spray cooling increased the critical heat flux up to 126% compared with water spray, and the Nusselt number and cooling efficiency were also significantly improved.^12^ Meanwhile, studies on borehole heat exchangers have indicated that excessive nanoparticle concentration may substantially increase viscosity and pressure drop, resulting in a system COP improvement of less than 1; in other words, the heat-transfer gain may be offset by increased pumping power consumption.^13^ Therefore, the key issue for nanofluid-assisted energy systems is not simply to “increase thermal conductivity” but to achieve a reasonable balance among heat-transfer enhancement, optical absorption, flow resistance, dispersion stability, and system energy consumption. These studies provide a broader engineering background for the present work. In thermoelectric refrigeration, electronic cooling, and geothermal heat exchange, nanofluids mainly improve system performance through convective heat-transfer enhancement, whereas in DASC the working fluid must directly absorb incident solar radiation; therefore, its optical absorption, photothermal conversion efficiency, and long-term dispersion stability are equally important. Based on this distinction, the heat-transfer-oil-based MWCNT-TiN hybrid nanofluid designed in this work does not primarily pursue high-concentration thermal-conductivity enhancement. Instead, it uses the broadband absorption of MWCNT and the enhanced visible–near-infrared absorption introduced by TiN at ppm-level concentrations to improve volumetric solar absorption while minimizing the viscosity increase, sedimentation risk, and pumping-power consumption that may occur in practical flow systems at high nanoparticle loadings.

To improve the solar absorption capability of nanofluids, researchers have widely explored dispersing carbon-based materials, metals, metal oxides, carbides, and nitrides into base fluids. Previous studies have shown that graphite/graphene,^14,15^ carbon nanotubes,^16,17^ gold,^18^ silver,^19^ CuO,^20,21^ Fe_3_O_4_,^22^ TiN,^23^ and other nanoparticles can improve the optical absorption and photothermal conversion performance of fluids to varying degrees. However, the solar spectrum consists of near-infrared, visible, and ultraviolet components,^24^ and mono nanofluids usually cannot sufficiently cover the entire solar spectrum. Therefore, their solar absorption performance is often limited.^25^ Hybrid nanofluids containing two or more types of nanoparticles can better match a broad spectrum and achieve stronger solar absorption, making their preparation an important route for improving solar collector performance.

Among carbon materials, MWCNT nanofluids show good application potential in DASC because of their high spectral absorptivity even at very low concentrations. Many researchers have prepared hybrid nanofluids with excellent photothermal performance by combining MWCNT nanoparticles with other nanoparticles. Tong et al^26^ investigated various MWCNT nanofluids in flat-plate solar collectors and found that the collector efficiency increased by 87%. Qu et al^27^ prepared water-based CuO–MWCNT hybrid nanofluids with significantly enhanced optical absorption; their results showed that the photothermal conversion performance of CuO–MWCNT hybrid nanofluids was generally superior to that of mono MWCNT nanofluids. Xuan and co-workers^28^ experimentally investigated the spectral absorption behavior of MWCNT–SiO_2_/Ag binary nanofluids and found that the binary nanofluid could absorb almost the entire solar spectrum. Li et al^25^ focused on the stability and photothermal conversion efficiency of SiC–MWCNT nanofluids with ethylene glycol (EG) as the base fluid. They reported that a 1 wt % SiC–MWCNT nanofluid achieved a photothermal conversion efficiency of 97.3%, which was 48.6% higher than that of the EG base fluid. Shin et al^29^ studied the thermal properties of MWCNT/Fe_3_O_4_ hybrid nanofluids and found that adding an appropriate amount of MWCNTs could enhance the photothermal performance of Fe_3_O_4_ nanofluids. Although these studies demonstrate that hybrid nanofluids can absorb solar energy more effectively than mono nanofluids, most related studies have focused on water-based systems. Heat-transfer-oil-based nanofluids have greater engineering potential for medium- and high-temperature solar-thermal utilization. Therefore, studying the photothermal conversion performance of MWCNT-TiN heat-transfer-oil-based nanofluids in DASC is important for developing efficient broadband-absorbing working fluids and expanding DASC engineering applications.

It should be noted that previous studies have already demonstrated that MWCNT-TiN hybrid nanofluids can improve DASC optical absorption and photothermal conversion performance through the broadband absorption of MWCNTs and the enhanced visible–near-infrared absorption introduced by TiN. For example, Zheng et al^30^ prepared water-based mono MWCNT and water-based MWCNT-TiN hybrid nanofluids and reported favorable photothermal conversion performance at a 10 ppm/10 ppm ratio. In addition, TiN is a non-noble-metal plasmonic material with an optical response in the visible–near-infrared region,^31^ and TiN nanoparticles have been reported to serve as effective solar-thermal transducers through broadband lossy plasmonic absorption.^32^ These studies provide background support for the role of TiN in photothermal enhancement, but they do not imply that the specific LSPR process can be directly confirmed solely from the steady-state UV–vis absorption spectra measured in the present work. Therefore, this study more cautiously describes the role of TiN as “broadband absorption enhancement after TiN incorporation” and treats LSPR as a possible mechanism consistent with previous literature rather than as the only or directly experimentally verified cause. Accordingly, this work does not claim the MWCNT-TiN hybrid absorption mechanism as a completely new material concept. Instead, it focuses on a heat-transfer-oil-based MWCNT-TiN hybrid nanofluid, which is more relevant to medium- and high-temperature solar-thermal applications. Compared with aqueous systems, heat-transfer oils have a higher applicable temperature range and better compatibility as high-temperature working fluids, but their low polarity changes the particle stabilization mechanism from the electric-double-layer repulsion commonly found in water-based systems to oil-phase wettability and steric stabilization. Thus, the differences and scientific contributions of the present work are mainly reflected in three aspects. (1) A heat-transfer oil, rather than water or ethylene glycol, is used as the base fluid for medium- and high-temperature DASC applications. This shift is not merely a medium replacement; it introduces fundamentally different physicochemical constraints, including a low-polarity environment that suppresses electrostatic stabilization, a higher viscosity that suppresses convective heat/mass transfer, and a lower specific heat capacity that alters the energy balance during photothermal conversion. (2) To overcome the stabilization challenge in this low-polarity oil phase, oleic-acid-assisted dispersion and ultrasonication are employed. Unlike aqueous systems where pH adjustment and electrostatic repulsion are effective, the oil-based system requires steric hindrance as the primary stabilization mechanism, and our results demonstrate its feasibility for MWCNT-TiN hybrids. (3) Under the same heat-transfer-oil baseline, the relationships among MWCNT concentration, TiN loading, spectral absorption, solar-weighted absorption fraction, temperature rise at different depths, and collector efficiency are systematically compared, revealing that the photothermal efficiency exhibits a nonmonotonic dependence on TiN concentration.

In this study, a heat-transfer-oil-based hybrid MWCNT-TiN nanofluid is proposed to expand the application of nanofluids in medium- and high-temperature DASC systems. First, the optical properties and photothermal conversion performance of mono MWCNT nanofluids with different mass fractions were analyzed to determine the optimal MWCNT concentration. Then, different proportions of TiN nanoparticles were introduced into the optimized MWCNT nanofluid to prepare MWCNT-TiN hybrid nanofluids. The optical and photothermal properties of the hybrid nanofluids were systematically investigated to further improve solar absorption and photothermal conversion efficiency through the combined effect of MWCNT and TiN light absorbers and to determine the optimal hybrid concentration for DASC applications. The results provide a theoretical basis and reference for selecting and applying hybrid nanofluids in medium- and high-temperature DASC.

## 2 Experimental Section

### 2.1 Materials and Preparation of Nanofluids

Mono MWCNT nanofluids and MWCNT-TiN hybrid nanofluids were prepared using a two-step method. The base oil was Therminol 66 heat-transfer fluid (Solutia/Eastman), a modified-terphenyl-based synthetic aromatic high-temperature liquid-phase heat-transfer oil. Its type, commercial name, and main thermophysical properties are listed in Table 1. For the MWCNT-TiN nanofluids, a certain amount of heat-transfer oil was first weighed using a high-precision electronic balance. Then, 0.05 wt % MWCNTs, 0.1 wt % oleic acid, and the required amount of TiN were added to the oil, the TiN addition ratios are summarized in Table 2. The suspension was stirred with a magnetic stirrer for 30 min and then ultrasonicated for 2 h in an ultrasonic cleaner. During ultrasonication, the nanofluid temperature was kept below 25 °C using a water bath. Finally, the nanofluid was diluted with heat-transfer oil to obtain different concentrations. The MWCNT nanofluids were prepared following a similar procedure but without adding TiN nanoparticles. The main preparation difference between the oil-based system in this work and previously reported water-based MWCNT-TiN systems is that the present work does not rely on aqueous pH adjustment or hydrophilic dispersants. Instead, oleic acid was used to improve particle wettability in the heat-transfer oil, and prolonged ultrasonication was employed to reduce initial agglomeration. This treatment is more suitable for dispersing hybrid particles in a low-polarity oil phase.

**Table 1:** Parameters and Values of the Base Oil

Parameter	Value
Base-oil type	Synthetic aromatic high-temperature liquid-phase heat-transfer oil; the main component is modified terphenyl
Commercial name/manufacturer	Therminol 66 heat-transfer fluid (Solutia/Eastman)
Density	1002 kg m^–3^ (30 °C); 955 kg m^–3^ (100 °C)
Specific heat capacity	1.60 kJ kg^–1^ K^–1^ (30 °C); 1.84 kJ kg^–1^ K^–1^ (100 °C)
Dynamic viscosity	55.6 mPa s (30 °C); 3.60 mPa s (100 °C)
Thermal stability/applicable temperature range	Recommended maximum bulk-fluid temperature of 345 °C; maximum film temperature of 375 °C; atmospheric boiling point of 359 °C; COC flash point of 184 °C
Optical properties	A clear light-yellow liquid. The UV–vis measurements in this work show that the average transmittance in the 300–800 nm range is approximately 68.43%, and the absorbance at 500 and 800 nm is approximately 0.06 and 0.02, respectively

**Table 2:** Concentrations of the Prepared Nanofluids

No.	C_MWCNT (ppm)	C_TiN (ppm)
1	10	0
2	20	0
3	30	0
4	10	5
5	10	10
6	10	15
7	10	20

To avoid simply assuming that the thermophysical properties of the nanofluids are identical to those of the base oil, the thermal conductivity, viscosity, density, and specific heat capacity of each sample at 30 °C were further listed. Because the nanoparticle concentrations in this work were only 5–30 ppm, the changes in thermophysical properties were very small. Therefore, commonly used effective-medium models were adopted: density and specific heat capacity were calculated using mass-fraction mixing, viscosity was estimated using the Brinkman model, and thermal conductivity was estimated using the Maxwell model. In the estimation, the density, specific heat capacity, and thermal conductivity of MWCNTs were taken as 2100 kg m^–3^, 0.71 kJ kg^–1^ K^–1^, and 3000 W m^–1^ K^–1^, respectively, while those of TiN were taken as 5220 kg m^–3^, 0.545 kJ kg^–1^ K^–1^, and 29 W m^–1^ K^–1^, respectively. It should be noted that, since the nanoparticle concentrations in this study are only 5–30 ppm (i.e., 0.0005–0.003 wt%), which fall into an extremely low concentration range, the changes in density, specific heat, viscosity, and thermal conductivity of the nanofluids compared to the base fluid are theoretically negligible. As can be seen from the estimation results in Table 3, the relative changes in all thermophysical properties of the nanofluid samples compared to the pure thermal oil are less than 0.02%. This magnitude of variation is far smaller than the systematic uncertainties of conventional experimental measurement instruments (for example, the measurement error of specific heat by DSC is typically within ±1%–3%, and that of viscosity is typically within ±1%–2%). Therefore, even if the thermophysical properties of the base fluid were directly used instead of the estimated values of the nanofluids in the efficiency calculation (eq 5), the ranking and comparison conclusions of the relative efficiencies among different working fluids would not be altered.

**Table 3:** Estimated Thermophysical Properties of the Base Oil and Nanofluids at 30 °C

Sample	Density (kg m^–3^)	Specific heat (kJ kg^–1^ K^–1^)	Viscosity (mPa s)	Thermal conductivity (W m^–1^ K^–1^)
Therminol 66	1002.000	1.60000	55.6000	0.117200
10 ppm MWCNT	1002.005	1.59999	55.6007	0.117202
20 ppm MWCNT	1002.010	1.59998	55.6013	0.117203
30 ppm MWCNT	1002.016	1.59997	55.6020	0.117205
10 ppm MWCNT + 5 ppm TiN	1002.009	1.59999	55.6008	0.117202
10 ppm MWCNT + 10 ppm TiN	1002.013	1.59998	55.6009	0.117202
10 ppm MWCNT + 15 ppm TiN	1002.017	1.59998	55.6011	0.117203
10 ppm MWCNT + 20 ppm TiN	1002.021	1.59997	55.6012	0.117203

### 2.2 Solar Thermal Conversion Experimental System

Figure 1 shows the schematic diagram of the experimental system used to evaluate the photothermal conversion performance of mono MWCNT and MWCNT-TiN hybrid nanofluids. The nanofluid was placed in a glass container with an inner diameter of 40 mm, a height of 15 mm, and a wall thickness of 2 mm. To reduce heat loss during irradiation, the container was covered with polystyrene foam (thermal conductivity as low as 0.03 W m^–1^ K^–1^), and a thin quartz glass sheet was placed on the top. A 300 W xenon-lamp solar simulator equipped with an AM1.5 filter (PLS-SXE300/300UV) was used to irradiate the nanofluids. During the experiment, the solar irradiance was measured using an irradiance meter (CEL-NP2000, Ceaulight) and maintained at 1000 W m^–2^. Three thermocouples were installed at different depths in the collector to monitor the temperatures of the top, middle, and bottom fluid layers. All temperatures were recorded every 2 s using a data acquisition unit (349720A, Keysight) and transmitted to a computer.

**Figure 1:**
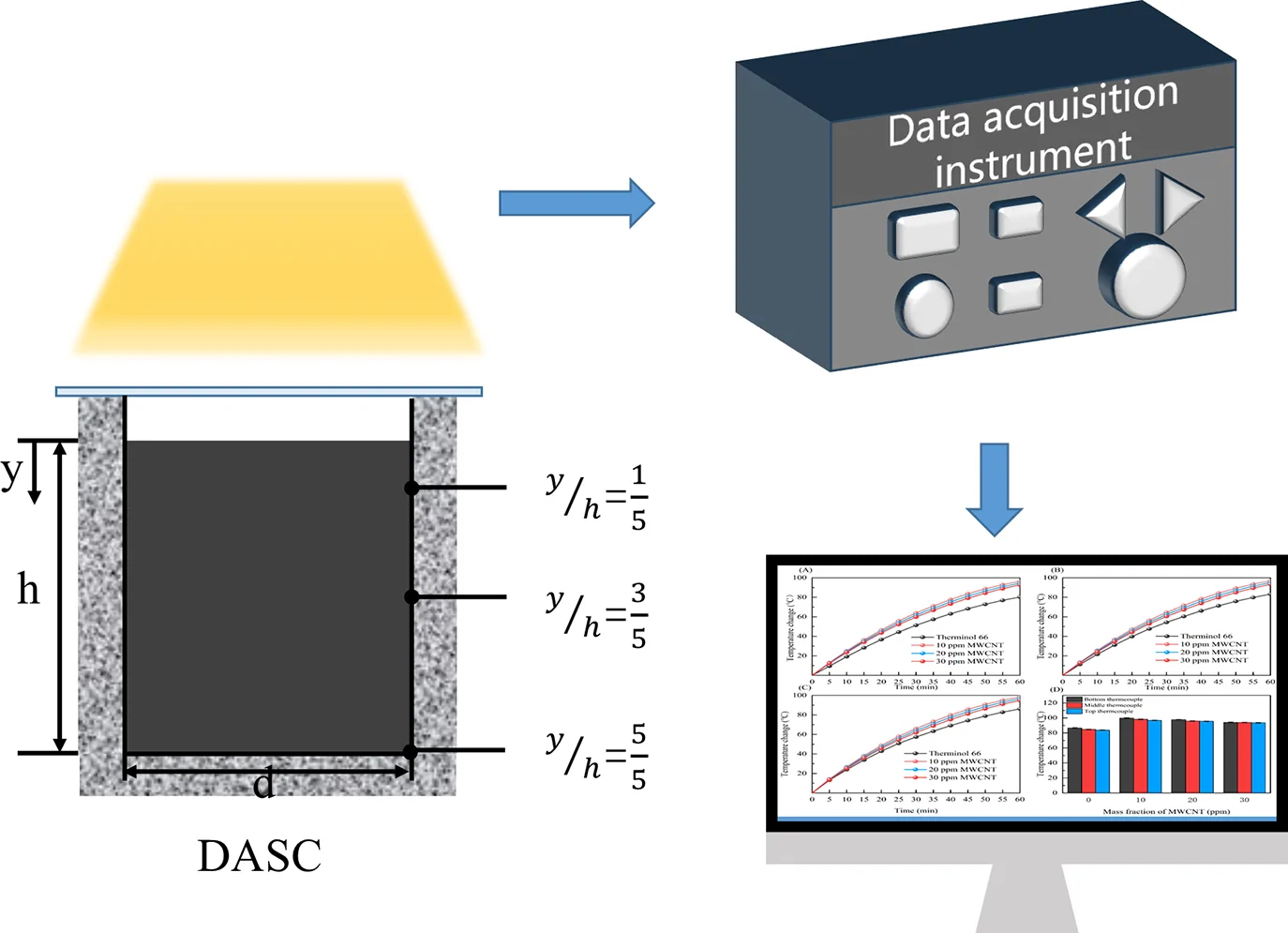
Photothermal conversion experimental system.

### 2.3 Experimental Methods

#### 2.3.1 Optical Properties

The optical transmission spectrum T­(λ) and absorbance spectrum A­(λ) of the nanofluid are presented as follows:

1 $$T\left(\lambda \right)=\frac{I\left(\lambda \right)}{I_{0}\left(\lambda \right)}$$

2 $$A\left(\lambda \right)=\log_{10}I_{0}\left(\lambda \right)/I\left(\lambda \right)=\log_{10}1/T\left(\lambda \right)$$

where I0 and I are the intensities of the incident light and the light transmitted through the nanofluid, respectively. Equation 2 does not separately account for scattering and is valid only when the scattering component is less than 10% of the total extinction. According to Mie scattering theory,^33^ scattering is mainly affected by the particle size and concentration in the nanofluid. In this study, the MWCNTs (inner diameter 5–10 nm, outer diameter 10–20 nm, length 10–30 μm) and TiN nanoparticles (20 nm) were used at relatively low concentrations (not exceeding 30 ppm), such that the investigated nanofluids were in the independent-scattering regime.^34^ Therefore, radiative scattering was neglected in this study.

The solar-weighted absorption fraction (F) represents the percentage of incident solar energy absorbed by the fluid at a given penetration depth and is another key parameter for evaluating nanofluids used in DASC. The solar-weighted absorption fraction is defined as:^35^

3 $$F=\frac{\int_{\lambda_{\min}}^{\lambda_{\max}}I_{0}\left(\lambda \right)\left(1-e^{-\alpha \lambda y}\right)d\lambda }{\int_{\lambda_{\min}}^{\lambda_{\max}}I_{0}\left(\lambda \right)d\lambda }$$

where α­(λ)^36^ is the extinction coefficient of the nanofluid, which is determined according to the Beer–Lambert law:

4 $$T\left(\lambda \right)=e^{-\alpha \left(\lambda \right)y}$$

where y represents the penetration depth.

#### 2.3.2 Photothermal Conversion Efficiency

The photothermal conversion efficiency of the collector is expressed as:^23^

5 $$\eta =\frac{{\mathrm{mc}}_{P}\left(T_{\mathrm{bulk}}-T_{\mathrm{initial}}\right)}{\mathrm{GA}\Delta t}=\frac{\mathrm{hp}c_{P}\left(T_{\mathrm{bulk}}-T_{\mathrm{initial}}\right)}{G\Delta t}$$

where *m*, ρ, and cp represent the mass, density, and specific heat capacity of the nanofluid, respectively. In eq 5, A is the top illuminated area of the collector and h is the height of the nanofluid. *T_bulk* is the bulk temperature of the nanofluid, defined as the arithmetic mean temperature at different fluid depths, *T_initial* is the initial temperature, and *G* and *Δt* are the solar irradiance and irradiation time, respectively.

Equation 5 calculates the photothermal conversion efficiency without heat-loss correction. It essentially represents the ratio of the sensible heat stored in the fluid over a given irradiation time to the incident solar energy and does not explicitly include convective, radiative, or conductive heat losses between the container wall, the top quartz glass, and the surrounding environment. In addition, the estimated thermal conductivity, viscosity, density, and specific heat capacity values of each nanofluid sample are provided in Table 3. The corresponding *ρ_nf* and *c_p, nf* values of each sample were used in the efficiency calculation. Because the nanoparticle loading was at the ppm level, the differences between *ρ_nf* and *c_p, nf* and those of the base oil were very small; therefore, this treatment did not alter the relative efficiency trends among different working fluids.

According to eq 5, the photothermal conversion efficiency is affected by the specific heat capacity of the nanofluid, temperature variation, solar irradiance, and irradiation time. Therefore, the relative uncertainty *U_η* of the photothermal conversion efficiency can be determined as follows:

6 $$U_{\eta }=\sqrt{{\left(\frac{U\mathrm{cp}}{\mathrm{cp}}\right)}^{2}+\overset{3}{\underset{i=1}{\sum }}{\left(\frac{U\Delta T_{i}}{\Delta T_{i}}\right)}^{2}+{\left(\frac{UG}{G}\right)}^{2}+{\left(\frac{U\Delta t}{\Delta t}\right)}^{2}}$$

Based on the accuracy of the experimental instruments and recording conditions, the thermocouple/data acquisition temperature uncertainty was taken as ±0.5 °C, the irradiance fluctuation of the solar simulator as ±3.0%, and the uncertainties of irradiation time and base-oil specific heat capacity as ±0.03% and ±1.0%, respectively. Using a smaller temperature rise of ΔT ≈ 40 K in the photothermal conversion experiment as a conservative condition, the maximum relative uncertainty of the photothermal conversion efficiency was approximately ±3.7%. Therefore, the experimental data in this study are acceptable.

## 3 Morphology of Nanoparticles and Stability of Nanofluids

### 3.1 Morphology and Structure of Nanoparticles and Dispersion of Nanofluids

Scanning electron microscopy (SEM) was used to observe the micromorphology of MWCNT and TiN nanoparticles. As shown in Figure 2, the MWCNT nanoparticles exhibited a tubular morphology, whereas the TiN nanoparticles were approximately spherical. Figure 3 shows the transmission electron microscopy (TEM) images of the MWCNT, TiN, and hybrid MWCNT-TiN nanofluids. The MWCNT and TiN nanoparticles were well dispersed, indicating that they are suitable for nanofluid preparation.

**Figure 2:**
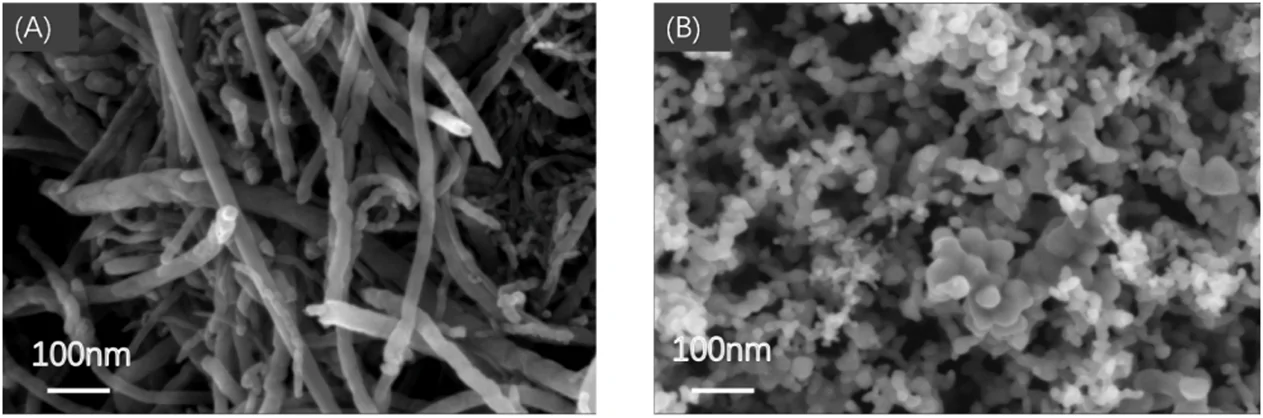
SEM images of (A) MWCNT and (B) TiN nanoparticles.

**Figure 3:**
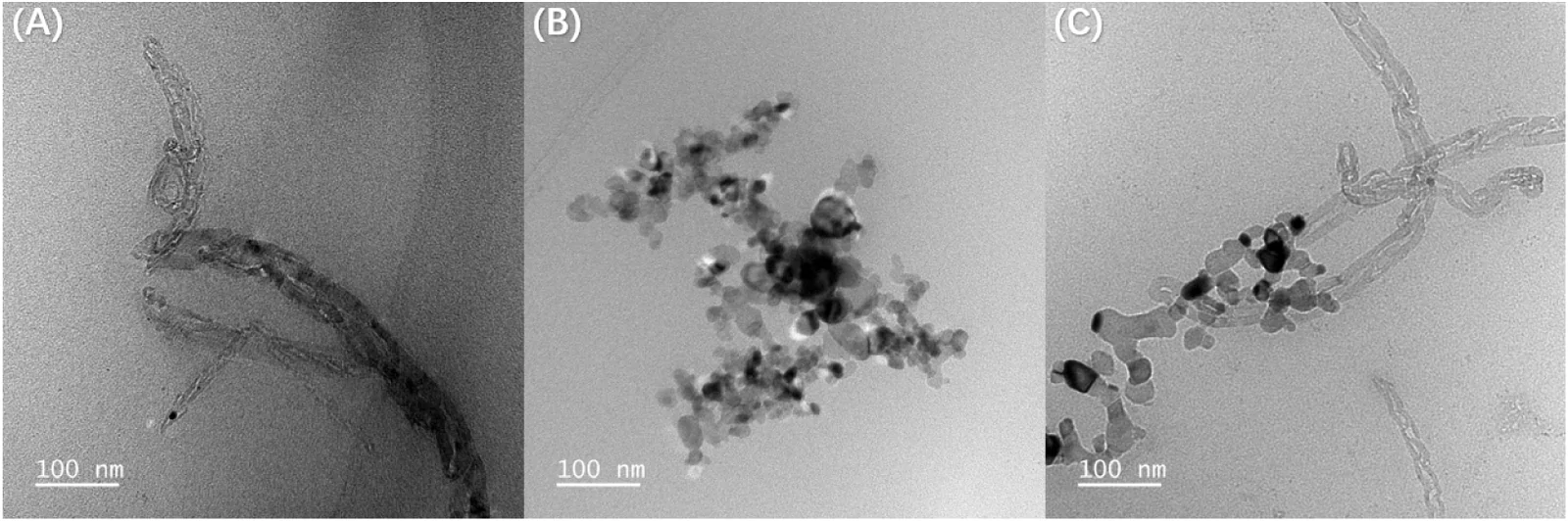
TEM images of (A) MWCNT, (B) TiN, and (C) hybrid MWCNT-TiN nanofluids.

### 3.2 Nanofluid Stability

The dispersion stability of nanofluids is essential for maintaining continuous light absorption and stable photothermal conversion when they are used as DASC working fluids. Insufficient stability may cause particle agglomeration, sedimentation, and local concentration gradients, resulting in a decrease in the absorption coefficient and photothermal conversion efficiency over time. Recent studies commonly evaluate nanofluid stability using visual sedimentation, UV–vis absorbance retention, DLS particle-size distribution, and zeta potential. Among these methods, |ζ| ≈ 30 mV is often regarded as a basic stability threshold for water-based or polar-base-fluid nanofluids, while |ζ| values above 45 mV generally indicate stronger electrostatic repulsion and better stability.^8^ Meanwhile, surfactant addition, pH adjustment, particle functionalization, and ultrasonication are considered the main strategies for reducing agglomeration risk.^8,37,38^

Compared with the stability criteria mainly established for water-based systems, the heat-transfer-oil-based MWCNT-TiN hybrid nanofluid in this work has a different stabilization mechanism. Heat-transfer oil is a low-polarity base fluid, in which electric-double-layer repulsion is relatively weak; therefore, evaluating its stability solely using the zeta-potential threshold commonly applied to water-based nanofluids has certain limitations. For such oil-based systems, particle surface wettability in the oil phase and steric hindrance are more critical. In this work, oleic acid was used as an oil-soluble dispersant and combined with 2 h ultrasonication, which helps improve the compatibility between MWCNT/TiN surfaces and the heat-transfer oil. In addition, the nanoparticle loading in this study was at the ppm level, much lower than the 0.1–2 wt % or higher concentration ranges commonly used in many heat-transfer nanofluid studies. This low particle concentration helps reduce the probability of collision-induced agglomeration and sedimentation.^37^

As shown in Figure 4A, neither the mono MWCNT nanofluids nor the hybrid MWCNT-TiN nanofluids showed obvious phase separation or visible sedimentation after standing for 7 d. Furthermore, the UV–vis absorption spectra in Figure 4B indicate that the absorption curves of the two types of nanofluids before and after 7 d of standing were generally consistent, with only slight fluctuations. This suggests that the effective particle concentration in the upper-layer sample changed only slightly during storage. These results indicate that, within the short term DASC photothermal testing period considered in this work, the heat-transfer-oil-based MWCNT-TiN system can maintain good macroscopic dispersion stability and optical absorption stability.

**Figure 4:**
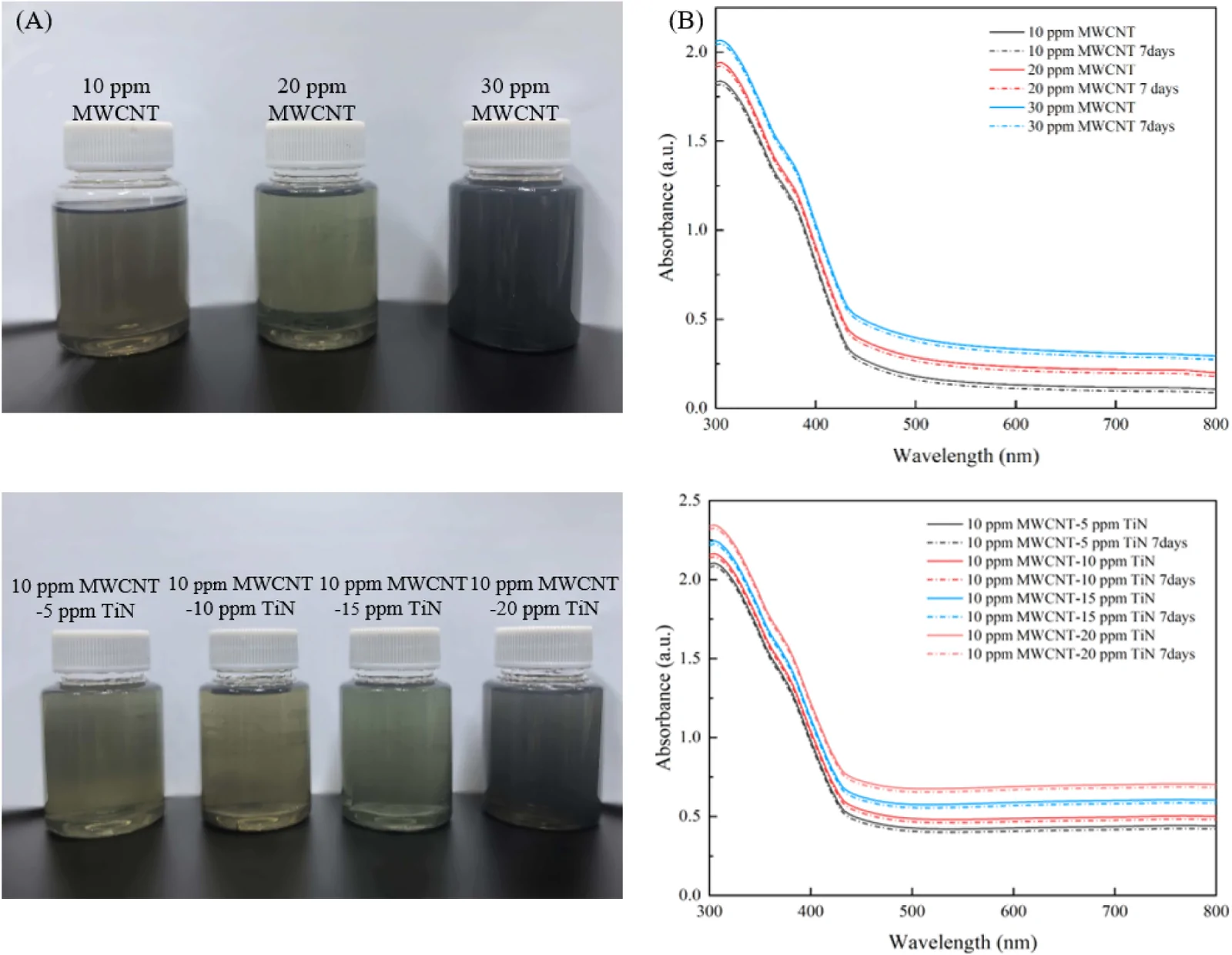
Photographs of MWCNT and hybrid MWCNT-TiN nanofluids after 7 d of standing (A), and comparison of the absorption spectra of MWCNT and hybrid MWCNT-TiN nanofluids before and after 7 d of standing (B).

## 4 Analysis of the Photothermal Properties of Three Thermal Conductive Oil-Based Multiwalled Carbon Nanotube Nanofluids

### 4.1 Optical Properties of Multiwalled Carbon Nanotube Nanofluids

The optical properties of MWCNT nanofluids are shown in Figure 5. For DASC applications, nanofluids should have low transmittance and high absorbance. As shown in Figure 5A, the addition of MWCNTs significantly increased the absorbance of the nanofluid in the near-infrared–visible wavelength range. At 500 nm, the absorbance of the 10 ppm MWCNT nanofluid was 0.18, approximately three times that of the heat-transfer oil. At 800 nm, the absorbance of the heat-transfer oil was 0.02, whereas that of the 10 ppm MWCNT nanofluid reached 0.12. This indicates that MWCNTs markedly improved the absorption performance compared with the base fluid. As shown in Figure 5B, the transmittance of the MWCNT nanofluid gradually decreased with increasing mass fraction. At 10 ppm, the average transmittance of the nanofluid was approximately 53.18%, whereas at 30 ppm it decreased significantly to 33.44%. The transmittance is expected to continue decreasing with further increases in nanoparticle concentration. The extinction coefficient shown in Figure 5C followed the same trend as the absorption spectrum. This is because, when the nanoparticles are small and well dispersed, the effect of scattering is minor, and the measured extinction coefficient is approximately equivalent to the absorption coefficient. The solar-weighted absorption fraction is a key parameter for evaluating the absorption and energy-storage capability of nanofluids. Figure 5D shows that, at the same penetration depth, MWCNT nanofluids exhibited much stronger solar energy absorption than Therminol 66. Samples with mass fractions above 10 ppm could absorb almost all the incident energy within a 10 cm fluid depth, and the required full-absorption depth decreased as the mass fraction increased.

**Figure 5:**
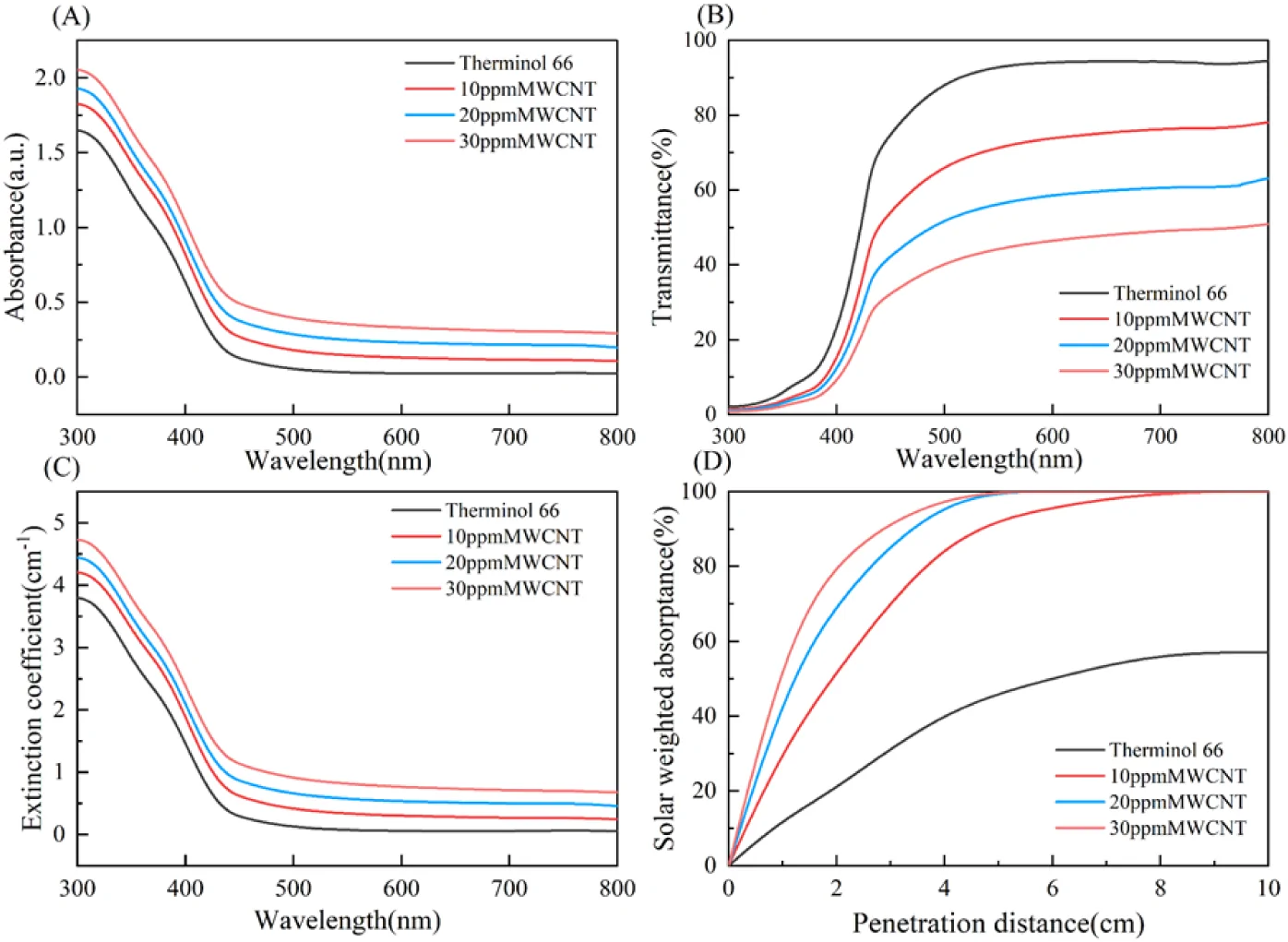
Absorption spectra (A), transmittance spectra (B), extinction coefficient (C), and solar-weighted absorption fraction (D) of MWCNT nanofluids at different concentrations.

### 4.2 Temperature Distribution of Multiwalled Carbon Nanotube Nanofluids

As shown in Figure 6A–C, the temperatures of all working fluids at the bottom, middle, and top measurement positions increased with irradiation time and eventually tended to stabilize. Compared with the base fluid, the MWCNT nanofluids exhibited higher temperature rises. Under the present experimental conditions, the 10 ppm MWCNT sample achieved the highest temperature rise at the bottom position after 60 min of solar irradiation, reaching 98.3 °C, whereas the base fluid reached only 85.1 °C, which was 14.2 °C lower than that of the MWCNT nanofluid. To further compare the effect of mass fraction, Figure 6D shows the temperature changes at the bottom, middle, and top fluid layers as a function of MWCNT mass fraction. After 60 min of irradiation, the temperature change of the MWCNT nanofluid increased slightly with increasing MWCNT mass fraction and reached a maximum at 10 ppm. Further increasing the mass fraction did not lead to additional temperature enhancement. This is because, at higher mass fractions, incident radiation is more readily absorbed by the upper fluid layer, reducing the effective absorption depth and increasing the local temperature gradient; therefore, further increases in particle concentration cannot continue to improve the measurable bulk sensible heat storage.

**Figure 6:**
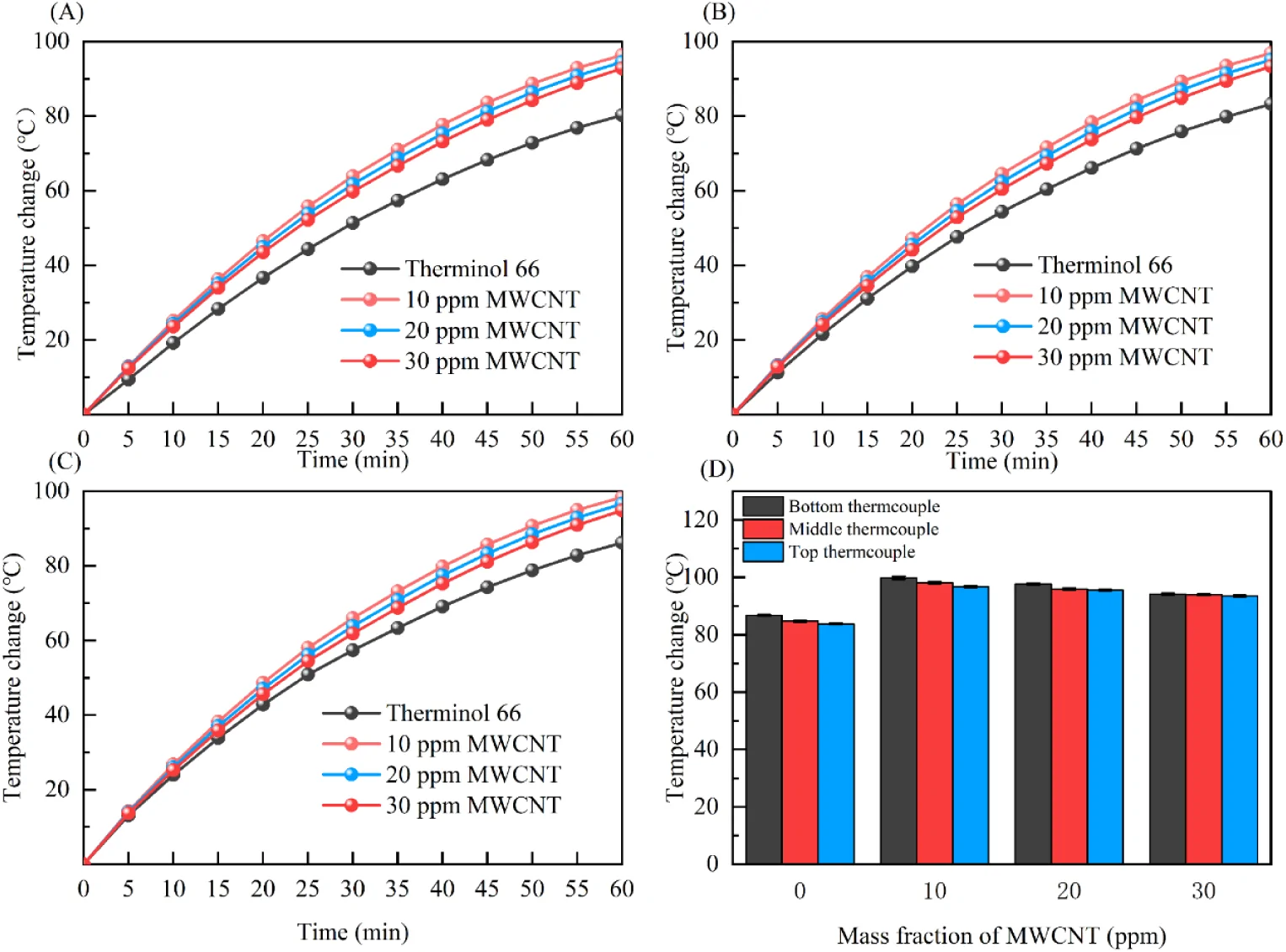
Temperature variations of the bottom fluid (A), middle fluid (B), and top fluid (C) with irradiation time, and comparison of the temperature changes in the top, middle, and bottom fluid layers after 60 min of irradiation for MWCNT nanofluids at different mass fractions (D).

### 4.3 Photothermal Conversion Efficiency of Multiwalled Carbon Nanotube Nanofluids

Figure 7A shows the photothermal conversion efficiency of the MWCNT nanofluids as a function of time. Overall, the collector efficiency of all working fluids gradually decreased with irradiation time. Compared with the base fluid, the MWCNT nanofluids significantly improved the collector efficiency. The 10 ppm sample showed the most obvious enhancement, increasing the maximum efficiency from 64.70% to 82.51%. When the MWCNT mass fraction was further increased, the efficiency differences among nanofluids with different concentrations became small, indicating that a low mass fraction is sufficient to significantly improve collector efficiency. Figure 7B compares the efficiencies of the MWCNT nanofluids and the base fluid at 20, 40, and 60 min. At all irradiation times, the efficiency of the 10 ppm MWCNT nanofluid was higher than those of the base fluid and the higher-concentration nanofluids, reaching 68.46%, 53.5%, and 40.06% at 20, 40, and 60 min, respectively. Compared with the base fluid, the efficiency improvements of the MWCNT nanofluid were 14.5%, 10.3%, and 9.4%, respectively. Therefore, the 10 ppm MWCNT nanofluid was selected as the basis for constructing the subsequent MWCNT-TiN hybrid nanofluids.

**Figure 7:**
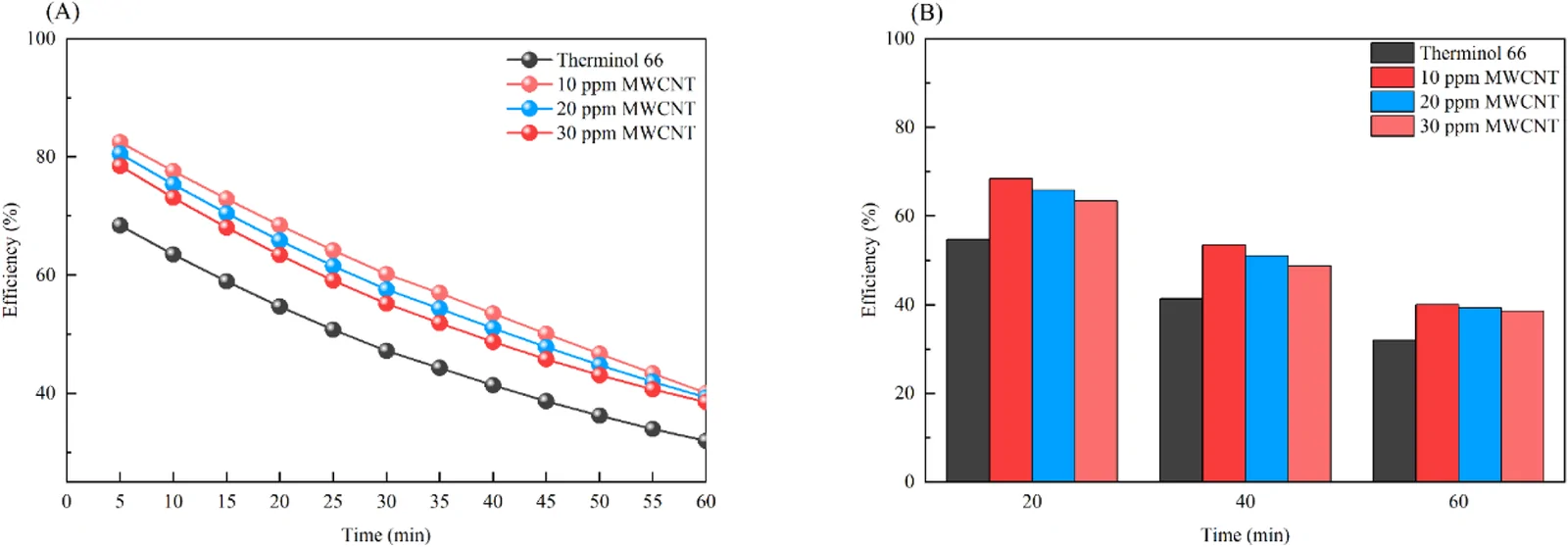
Collector efficiency of MWCNT nanofluids at different mass fractions as a function of irradiation time (A), and comparison of collector efficiency after 60 min of irradiation (B).

## 5 Analysis of the Photothermal Properties of MWCNT-TiN Composite Nanofluids

### 5.1 Optical Properties of MWCNT-TiN Composite Nanofluid

The optical properties of MWCNT-TiN hybrid nanofluids at different mass fractions are shown in Figure 8. As shown in Figure 8A–C, compared with the heat-transfer oil base fluid and the mono 10 ppm MWCNT nanofluid, the addition of TiN resulted in higher absorbance, lower transmittance, and a larger extinction coefficient in the near-infrared–visible range. At 500 nm, the absorbance of the 10 ppm MWCNT–10 ppm TiN sample was 0.48, which was significantly higher than that of the 10 ppm MWCNT nanofluid. At 800 nm, the absorbance of the 10 ppm MWCNT nanofluid was 0.11, whereas that of the 10 ppm MWCNT–10 ppm TiN sample increased to 0.52, with the transmittance decreasing to approximately 31%. As the TiN mass fraction increased from 5 ppm to 20 ppm, the absorbance and extinction coefficient of the hybrid nanofluid generally increased, indicating that TiN incorporation indeed enhanced the spectral extinction capability in the visible–near-infrared region. Figure 8D further shows that adding TiN nanoparticles increased the solar-weighted absorption fraction. When the TiN mass fractions were 5, 10, 15, and 20 ppm, the solar-weighted absorption fractions at a 2 cm penetration depth reached 91.65%, 94.89%, 98.65%, and 99.9%, respectively, which were significantly higher than the 62.38% of the 10 ppm MWCNT nanofluid. Mechanistically, MWCNTs provide continuous broadband absorption, whereas TiN mainly raises the absorption baseline and extinction coefficient of the hybrid system in the visible–near-infrared region. This result is consistent with previous reports that TiN exhibits plasmonic optical responses in the visible–near-infrared region and can serve as a solar-thermal conversion material,^31,32^ although the present steady-state UV–vis measurements do not directly establish the plasmonic resonance mechanism.

**Figure 8:**
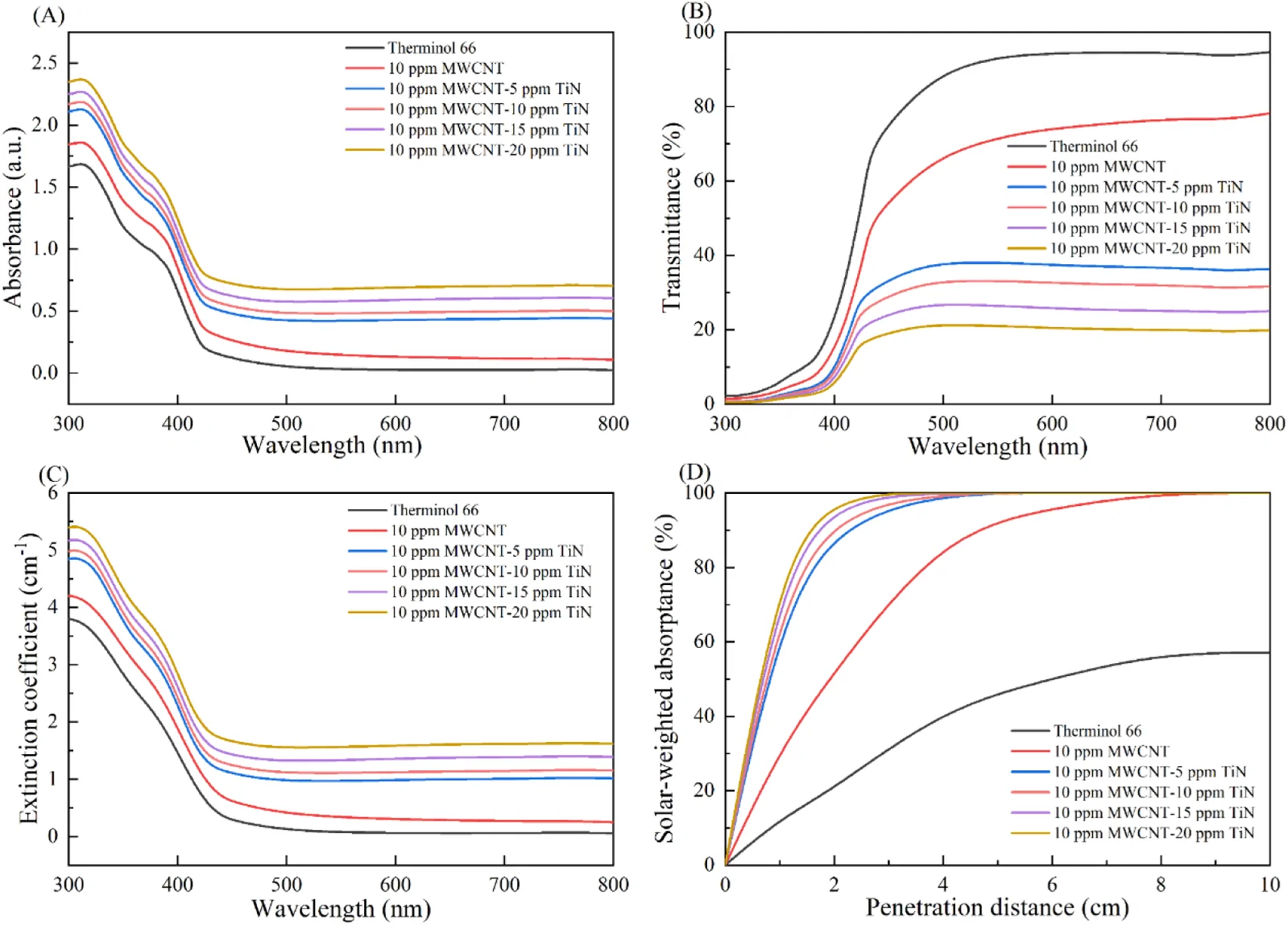
Absorption spectra (A), transmittance spectra (B), extinction coefficient (C), and solar-weighted absorption fraction (D) of MWCNT-TiN hybrid nanofluids at different mass fractions.

### 5.2 Temperature Distribution of MWCNT-TiN Composite Nanofluid

Figure 9A–C shows the temperature variations with irradiation time at the bottom, middle, and top positions of the collector for the MWCNT-TiN hybrid nanofluids, mono MWCNT nanofluid, and base fluid. The temperatures of all three working fluids increased with irradiation time and eventually tended to stabilize, and the temperature rises of the MWCNT-TiN hybrid nanofluids and mono MWCNT nanofluid were significantly higher than that of the base fluid. Notably, the temperature rise of the MWCNT-TiN hybrid nanofluid was slightly higher than that of the 10 ppm mono MWCNT nanofluid, indicating that the photothermal conversion performance of the hybrid system was further improved. To further analyze the effect of TiN mass fraction, Figure 9D shows the temperature rise at the top, middle, and bottom fluid layers of the MWCNT-TiN hybrid nanofluids with different TiN mass fractions after 60 min of irradiation. The temperature rises of the hybrid nanofluids with different TiN contents were relatively close, suggesting that, under the present experimental conditions, the TiN mass fraction had only a small effect on the temperature change.

**Figure 9:**
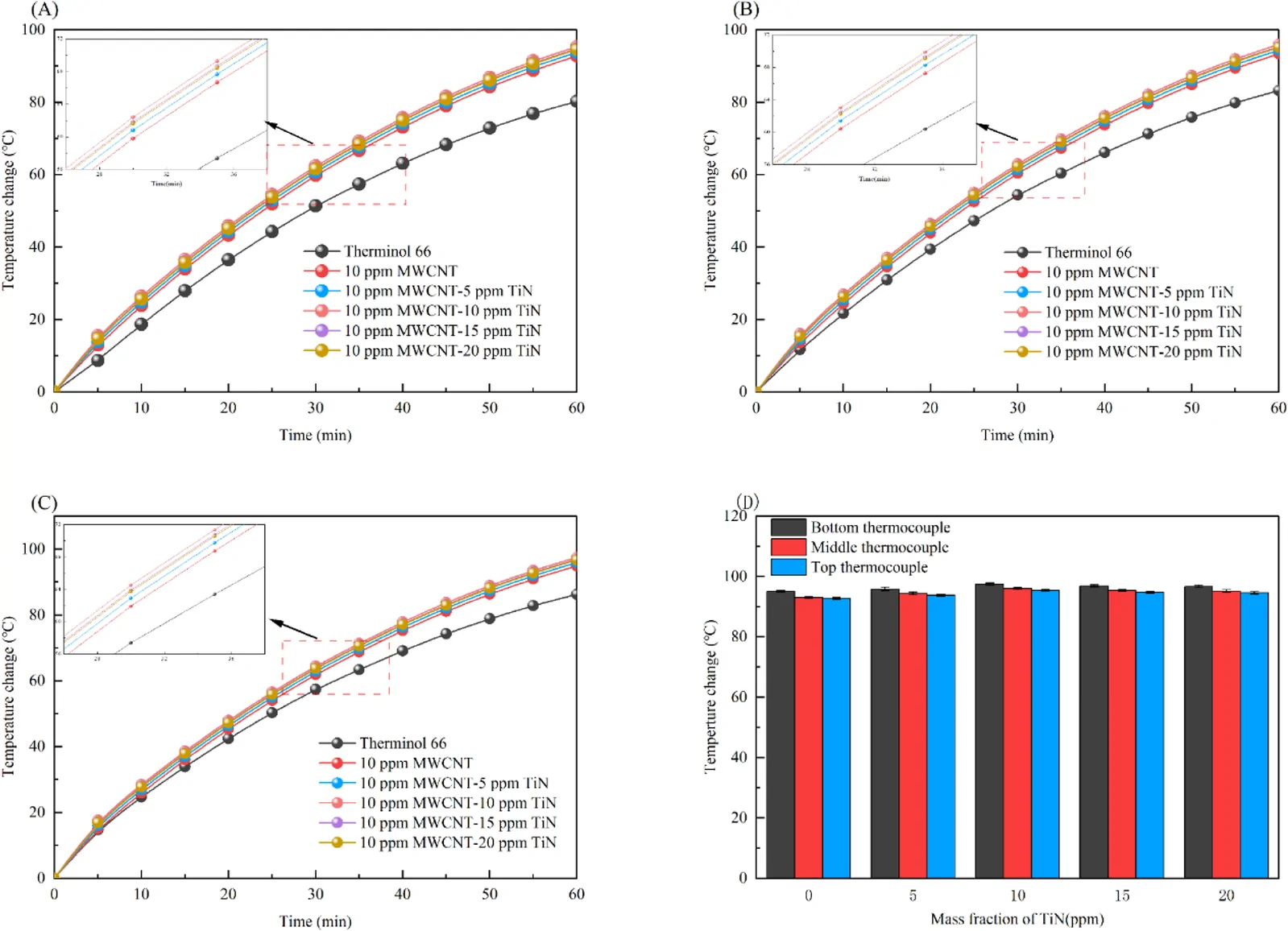
Temperature variations with irradiation time of the bottom fluid (A), middle fluid (B), and top fluid (C) for MWCNT-TiN hybrid nanofluids, and comparison of the temperature changes in the top, middle, and bottom fluid layers after 1 h of irradiation for hybrid MWCNT-TiN nanofluids with different TiN mass fractions (D).

### 5.3 Photothermal Conversion Efficiency of MWCNT-TiN Composite Nanofluid

Figure 10A compares the collector efficiencies of the MWCNT-TiN hybrid nanofluids, MWCNT nanofluid, and base fluid. As the irradiation time increased, the collector efficiencies of the base fluid, mono MWCNT nanofluid, and hybrid MWCNT-TiN nanofluids with different TiN contents all gradually decreased. This decreasing trend should be understood from the efficiency definition in eq 5: as the system temperature increased and gradually approached thermal equilibrium, the fluid temperature-rise rate decreased, and the rate of cumulative sensible-heat growth became lower than the rate of cumulative incident-energy input, leading to a decrease in efficiency. Figure 10B further compares the efficiencies of different samples at 20, 40, and 60 min. The efficiencies of the MWCNT-TiN hybrid nanofluids were higher than those of the pure heat-transfer oil and mono MWCNT nanofluid, among which the 10 ppm MWCNT–10 ppm TiN sample showed the highest efficiency. Compared with the 10 ppm MWCNT nanofluid, the apparent efficiencies of the 10 ppm MWCNT–10 ppm TiN sample increased by 7.9%, 5.4%, and 3.8% at 20, 40, and 60 min, respectively. This indicates that, under the present experimental geometry and optical path length, the optimal TiN content is jointly determined by absorption intensity and volumetric heating uniformity and cannot be judged solely from the magnitude of the solar-weighted absorption fraction.

**Figure 10:**
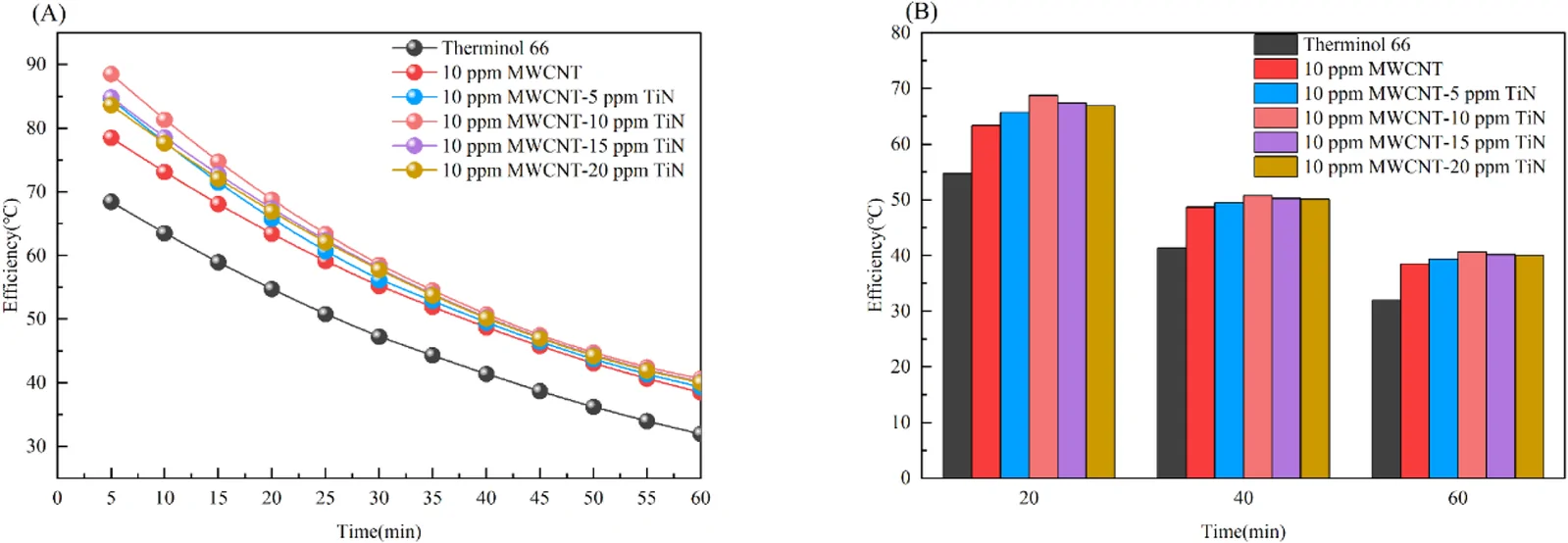
Collector efficiency of MWCNT-TiN hybrid nanofluids with different TiN concentrations as a function of irradiation time (A), and comparison of collector efficiency after 60 min of irradiation (B).

### 5.4 Stability Evaluation of the Optimized MWCNT-TiN Hybrid Nanofluid

Figure 11A shows the UV–vis absorption spectra of the optimized 10 ppm MWCNT–10 ppm TiN hybrid nanofluid after 0, 10, and 20 thermal cycles. The three curves showed nearly identical overall shapes. Strong absorption was maintained in the 300–400 nm region, and no obvious baseline decrease or new abnormal peak appeared in the 450–800 nm region. As the number of thermal cycles increased, only a slight decrease in absorbance was observed, indicating that repeated heating/cooling did not significantly damage the optical absorption capability of the MWCNT-TiN hybrid nanofluid. Specifically, the absorbance retention after 10 cycles was approximately 98.0%, and that after 20 cycles remained approximately 95.5%, indicating good short-term thermal-cycling optical stability.

**Figure 11:**
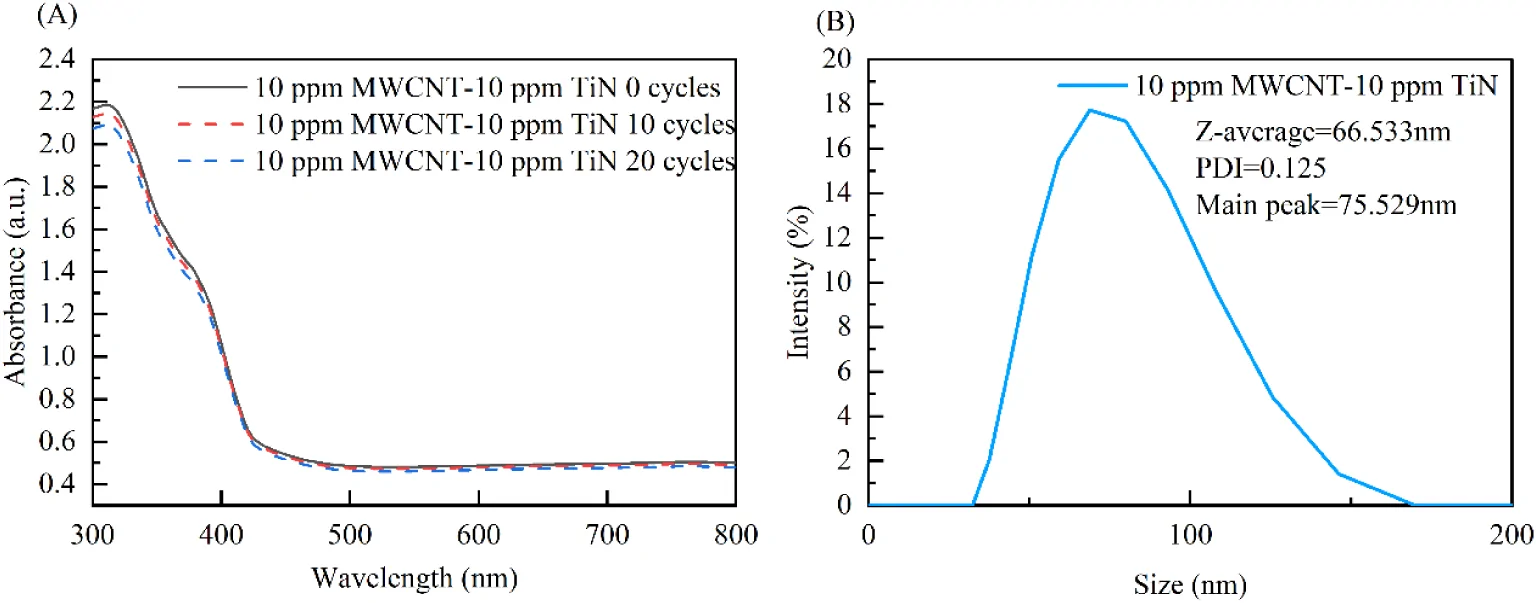
Optical absorbance retention (A) and particle-size distribution (B) of the optimized 10 ppm MWCNT–10 ppm TiN oil-based hybrid nanofluid after thermal cycling.

Figure 11B shows the particle-size distribution of the optimized hybrid nanofluid. The sample exhibited a single-peak distribution, with the main peak located at approximately 75.529 nm, a Z-average of 66.533 nm, and a PDI of 0.125. A PDI lower than 0.2 indicates a relatively narrow particle-size distribution and good dispersion uniformity. In addition, no large-particle peak above 200 nm or in the micrometer range appeared in the figure, indicating that no obvious large-size aggregates were present in the sample. Therefore, the particle-size distribution results support that the optimized MWCNT-TiN hybrid nanofluid had a good dispersion state after oleic-acid-assisted dispersion and ultrasonication.

## 6 Conclusions

In this work, mono MWCNT nanofluids and MWCNT-TiN hybrid nanofluids were prepared by a two-step method using Therminol 66 heat-transfer oil as the base fluid. Their dispersion stability, optical absorption characteristics, temperature response, and apparent photothermal conversion performance were systematically investigated. Compared with previous water-based MWCNT-TiN studies, this work extends the hybrid absorber to a low-polarity heat-transfer oil medium and further evaluates the thermal-cycling optical stability and particle-size distribution of the optimized sample. The main conclusions are as follows:

1. The addition of MWCNTs significantly enhanced the solar radiation absorption capability of Therminol 66 heat-transfer oil. As the MWCNT concentration increased from 10 ppm to 30 ppm, the average transmittance of the nanofluid decreased from 68.43% to 33.44%. Considering the optical properties, temperature rise, and apparent collector efficiency, the 10 ppm MWCNT nanofluid exhibited the best overall performance under the present experimental conditions and was therefore selected as the basis for constructing the MWCNT-TiN hybrid nanofluids.
2. Introducing TiN nanoparticles into the 10 ppm MWCNT nanofluid further enhanced the absorption and extinction capabilities of the hybrid system in the visible–near-infrared region. When the TiN concentrations were 5, 10, 15, and 20 ppm, the solar-weighted absorption fractions of the MWCNT-TiN hybrid nanofluids at a 2 cm penetration depth reached 91.65%, 94.89%, 98.65%, and 99.9%, respectively, all of which were significantly higher than that of the mono 10 ppm MWCNT nanofluid. This indicates that TiN incorporation can effectively enhance the visible–near-infrared extinction capability and volumetric solar absorption of the heat-transfer-oil-based hybrid nanofluid.
3. Among the different hybrid ratios, the 10 ppm MWCNT–10 ppm TiN hybrid nanofluid showed the highest apparent photothermal conversion efficiency, with a maximum efficiency of approximately 88.48%. Compared with the 10 ppm MWCNT mono nanofluid, its apparent efficiency increased by 7.9%, 5.4%, and 3.8% at irradiation times of 20, 40, and 60 min, respectively. It should be noted that although the 20 ppm TiN sample exhibited a higher solar-weighted absorption fraction, its efficiency did not further increase. This indicates that the optimal photothermal performance is not determined solely by maximum absorptivity. Instead, the advantage of the 10 ppm TiN sample arises from a balance between high absorption capability and an appropriate light penetration depth.
4. Supplementary stability evaluation of the optimized 10 ppm MWCNT–10 ppm TiN hybrid nanofluid showed that the overall UV–vis absorption spectra remained almost unchanged after 10 and 20 thermal cycles, with absorbance retentions of approximately 98.0% and 95.5%, respectively, indicating good short-term thermal-cycling optical stability. The particle-size distribution showed a single-peak profile, with a Z-average of 66.533 nm, a PDI of 0.125, and a main peak at 75.529 nm, and no obvious large-size aggregate peak was observed. These results further demonstrate that the optimized oil-based MWCNT-TiN hybrid nanofluid has good short-term dispersion stability and optical absorption stability.

## 7 Research Limitations and Future Work

It should be candidly acknowledged that this study has several limitations, the resolution of which will guide future research directions. First, regarding the experimental conditions, our original experimental design included a complete flow-loop test rig to evaluate the photothermal conversion performance and long-term cyclic stability of the nanofluids under controllable high-temperature conditions. However, due to the time constraint associated with the graduate student’s impending graduation, the construction and commissioning of the flow-loop test rig could not be completed as scheduled. Consequently, we resorted to a static stagnation irradiation experiment as an alternative in this work. Accordingly, the photothermal conversion efficiency reported herein is an apparent efficiency without heat-loss correction, and the convective, radiative, and conductive losses from the container walls, the top quartz glass, and the surrounding environment have not been precisely quantified. Meanwhile, the maximum fluid temperature reached in the static stagnation experiment was approximately 98.3 °C, which is far below the maximum operating temperature (345 °C) of Therminol 66 heat transfer oil. Therefore, the conclusions of this work are strictly applicable to performance validation and relative comparison under short-term, static, low-to-medium temperature (≤∼100 °C) conditions. Second, regarding material stability, although the oleic acid dispersant used in this study can maintain effective dispersing functionality within the experimental temperature range (≤∼100 °C), it may undergo thermo-oxidative decomposition at higher temperatures (>200 °C), and its long-term effectiveness under real medium-to-high temperature DASC operating conditions remains to be verified. In response to the above limitations, our research group has identified the following directions in the subsequent research plan: (1) complete the construction of the flow-loop test rig and evaluate the photothermal conversion efficiency of the nanofluids under high-temperature (>200 °C) conditions; (2) establish an accurate heat-loss correction model to convert the apparent efficiency into the net system efficiency; (3) conduct long-term continuous operation tests for thermal and dispersion stability, and explore high-temperature-resistant dispersants or particle surface functionalization modification strategies suitable for high-temperature oil-based systems. We are confident that the systematic resolution of the above issues will be accomplished step by step in our future work.
